# Innate Pathway Selection Modulates Antibody and T-Cell Responses to Mosaic Influenza Nucleoprotein in Cattle

**DOI:** 10.3390/v18060670

**Published:** 2026-06-13

**Authors:** Clara Cole, Thomas Cleven, Marlee Henige, Keith Poulsen, Mike Maroney, Lautaro Rostoll-Cangiano, Doerte Doepfer, Marulasiddappa Suresh

**Affiliations:** 1 Department of Pathobiological Sciences, University of Wisconsin-Madison, Madison, WI 53706, USA; 2 Department of Medical Sciences, University of Wisconsin-Madison, Madison, WI 53706, USA; 3 Wisconsin Veterinary Diagnostic Laboratory, University of Wisconsin-Madison, Madison, WI 53706, USA; 4 Research Animal Resources and Compliance, University of Wisconsin-Madison, Madison, WI 53706, USA; 5 Department of Animal and Dairy Sciences, University of Wisconsin-Madison, Madison, WI 53706, USA

**Keywords:** influenza virus, innate milieu, T-cell mediated immunity, adjuvants, T-cell epitope vaccine

## Abstract

Highly pathogenic avian influenza (HPAI) is a lethal disease of poultry that has recently spilled over into mammals, including dairy cattle and humans, heightening concerns for livestock health, food security, and pandemic emergence. While vaccines that induce neutralizing antibodies against hemagglutinin and neuraminidase provide strain-specific protection, durable cross-subtype immunity requires T-cell responses targeting conserved internal antigens such as nucleoprotein (NP). To leverage these conserved targets, we utilized a previously engineered mosaic nucleoprotein (MNP) incorporating T-cell epitopes from thousands of influenza A virus (IAV) strains, conferring broad protection against epidemic (H3N2) and pandemic (H1N1) IAV in mice. Here, we tested whether precision adjuvancy could differentially imprint adaptive immunity to MNP in cattle. Combination formulations paired the carbomer-based nano-emulsion Adjuplex (ADJ) with either a STING agonist (cyclic dinucleotides; CdN) or a TLR4 agonist (glucopyranosyl lipid A; GLA) to program distinct inflammatory milieus. Both formulations elicited circulating IFN-γ–producing T cell responses and NP-specific antibodies in serum and milk. However, STING activation via CdN generated more potent and consistent cellular and humoral immunity than TLR4 engagement. These data demonstrate that selective activation of innate sensing pathways functionally imprints adaptive immune magnitude and quality in a large animal host. By advancing a broadly protective, T-cell-focused vaccine strategy in cattle, this work supports a One Health framework to mitigate H5N1 transmission risk at the human–animal interface.

## 1. Introduction

Highly pathogenic avian influenza (HPAI) is caused by H5N1 influenza A virus, an enveloped RNA virus belonging to the *Orthomyxoviridae* family. HPAI is an exceedingly contagious disease that, when first discovered, appeared to only cause spontaneous death in birds. Between 1 October 2024 and 7 August 2025, there were over 5000 reports of H5N1 in wild birds and commercial and backyard poultry flocks worldwide [1]. Of the over 5000 cases, 3579 were reported in the United States [1]. Although originally thought to only cause disease in birds, since 2022, the USDA APHIS has reported HPAI H5N1 virus detections in over 200 mammalian species, including foxes, bears and seals, indicating mammalian spillover of this avian virus [2]. In March 2024, HPAI H5N1 virus was first detected in cattle; the virus detected was Influenza Virus A/Texas/37/2024 (H5N1) of the 2.3.4.4b clade and B3.13 genotype [2,3]. Since then, there have been close to 2000 reported affected dairy herds across 18 U.S. states, but the true number of positive farms is not known [4]. Clinical signs in dairy cattle vary and range from asymptomatic infection to mild febrility to severe pyrexia and mastitis [5]. Severe mastitis induced by H5N1 virus leads to markedly reduced milk production and quality and reduced feed intake [5]. Milk losses average 900 kg per cow for a duration of 60 days, which has an economic impact of ~$950 per cow [4,5]. This mammalian spillover and rapid onset of infection across dairy herds poses a health and economic risk to cattle and potentially other livestock and mammals, including humans.

RNA viruses such as influenza are highly prone to mutation due to the error-prone nature of their RNA-dependent RNA polymerase (RdRp), which lacks 3′–5′ proofreading-and-repair activity. These mutations can enhance receptor binding affinity, alter tissue tropism, or facilitate immune evasion, potentially leading to more severe clinical disease. Notably, the H5N1 bovine clade 2.3.4.4b has already evolved within a year of its initial detection, with the emergence of the D1.1 genotype reported in 2025 [6]. The D1.1 strain is more contagious, affects more mammalian species compared to previous variants, and has already caused 2 human deaths [6]. Although there is no evidence to date, the possibility of persistent H5N1 circulation in agricultural mammals and workers facilitates genetic evolution (e.g., through genetic reassortment), increasing pandemic potential and threatening the food industry. To limit disease severity and prevent transmission, we aimed to identify adjuvants that elicit robust and durable immune responses in cattle, thereby enabling the development of an effective vaccine against H5N1 and other influenza A strains.

To increase the effectiveness of vaccines, adjuvants are used. Adjuvants augment immunogenicity by either enhancing antigen delivery (e.g., alum, emulsions, liposomes, and polymeric particles) or potentiating the immune system (e.g., Toll-like receptor [TLR] agonists) [7]. Antibody and T-cell responses can be synergistically enhanced when both mechanisms are used [7]. Adjuplex (ADJ) is a nano-emulsion adjuvant that enhances the ability of dendritic cells (DCs) to process and present antigen to CD4 and CD8 T-cells due to its composition of Carbopol polymers and highly purified soy lecithin [8]. Previous studies have demonstrated that ADJ-based combination adjuvants can provide potent T-cell-based protection against infections, including SARS-CoV-2, influenza, and listeriosis [9,10,11]. Cyclic dinucleotides (CdN) and glucopyranosyl lipid A (GLA) are examples of adjuvants that potentiate the immune system by engaging two different pattern recognition pathways. CdN binds to and activates the STING pathway, which is triggered when the cell senses cytosolic DNA [12]. GLA is a synthetic TLR4 agonist which is typically activated by lipopolysaccharides (LPS) of Gram-negative bacteria [13]. Both CdN and GLA stimulate a cascade of events that result in production of pro-inflammatory cytokines and type I interferons, thus orchestrating a robust immune response. Studies have shown that CdN and GLA effectively enhance vaccine responses to influenza in mice, ferrets, and humans [14,15,16,17,18,19]. To our knowledge, the immunogenicity of the combination adjuvants ADJ + GLA and ADJ + CdN has not yet been evaluated in cattle.

While the influenza viral surface glycoproteins, hemagglutinin (HA) and neuraminidase (NA), are primary targets of virus-neutralizing antibodies, these responses are largely strain-specific. Additionally, antigenic drift in HA and NA facilitates immune escape. In contrast, T-cell responses directed against highly conserved internal proteins, such as nucleoprotein (NP), confer broad heterosubtypic immunity. During the 2009 swine-origin H1N1 pandemic, reduced disease severity was associated with pre-existing cross-reactive memory T-cells [20,21,22]. Leveraging NP sequences from thousands of influenza A virus (IAV) strains, we engineered a mosaic nucleoprotein (MNP) designed to maximize incorporation of conserved T-cell epitopes within the NP backbone. Notably, the MNP sequence exhibits high conservation with the recent bovine H5N1 isolate (Influenza A/Texas/37/2024) [23]. In mice, vaccination with MNP broadened T-cell responses and conferred superior protection against pandemic H1N1 compared with wild-type PR8 NP [23]. Although classical virus-neutralizing antibodies would not be induced, we chose MNP in this current formulation to elicit a strong T-cell-mediated immune response, which can provide broad heterosubtypic immunity. It is noteworthy that memory T-cells do not prevent infections, but promote accelerated viral clearance, thus limiting the duration of infection and protecting against severe clinical disease.

Here, we conducted a longitudinal study to evaluate the immunogenicity of MNP formulated with two combination adjuvants, ADJ + GLA and ADJ + CdN, in cattle. Both formulations induced robust antibody responses in serum and milk, as well as circulating T-cell responses. However, ADJ + CdN elicited more potent and consistent immunity than ADJ + GLA. Mechanistic studies in mice revealed that these two adjuvant combinations induced distinct innate cellular programs both systemically and within draining lymph nodes. Together, these findings demonstrate that selective engagement of innate sensing pathways functionally imprints the magnitude and quality of adaptive immunity in a large-animal host.

## 2. Materials and Methods

### 2.1. Experimental Animals

Fifteen three- to five-year-old female Holstein cattle in their first to fourth lactations were sourced from the Arlington Research Station or the Dairy Cattle Center (DCC) at the University of Wisconsin–Madison (WI, USA). Sample size was determined by the number of cattle available to be used for experimentation from the DCC. Prior to immunization, serologic testing was performed at the Wisconsin Veterinary Diagnostic Laboratory (WVDL; Madison, WI, USA), a Level 1 National Animal Laboratory Network (NAHLN) facility, accredited by the American Association of Veterinary Laboratory Diagnosticians. Screening included mastitis milk culture, Influenza A virus (IAV) matrix real-time PCR from nasopharyngeal swabs, and serum testing for leptospirosis, bovine herpesvirus, bovine viral diarrhea virus, bovine leukemia virus, and epizootic hemorrhagic disease. All results were negative or clinically insignificant. Groups of cattle were formed by stratified randomization, so that all cattle were female, lactating and three- to five-year-old. Cattle were then randomly assigned to groups so that all ages were represented in each group. Body weight, milk yield, feed intake, and temperature were monitored by DCC staff weekly, twice daily, daily, and on the day of vaccination and for 7 days thereafter, respectively. Cattle were all given the same feed and all measurements described above were taken at the same time to address potential confounders. All animals were housed in free stalls at the DCC, except for one from the ADJ + GLA group. Due to a physical injury on day 119, this animal was moved to loose housing at the Arlington Research Station to provide deeper bedding. One cow (from the ADJ + GLA group) had to be euthanized mid-study due to right hip dysplasia, unrelated to the experiment.

For the mouse experiment, eighteen C57BL/6J (B6) female mice aged 11–16 weeks were obtained from restricted-access, specific-pathogen-free breeding colonies at the University of Wisconsin–Madison Breeding Core Facility (WI, USA). Each group of mice was housed together in separate cages at the University of Wisconsin-Madison School of Veterinary Medicine Vivarium (WI, USA) and were acclimated for 3 days. All mice were provided the same bedding, feed, and water, kept in the same room on the same row of the cage rack, and were vaccinated and had tissues collected at the same time to address confounding variables. Sample size was determined prior to the experiment through Power analysis. Use of 5 mice per group, except for one group of 3 mice, yielded a power of 70–86% to detect large between-group differences (Cohen’s f ≥ 0.80) in cell composition at the one-way ANOVA level, consistent with the large effect sizes (f = 1.0–1.5+) typically observed for innate immune cell recruitment 48 h post-adjuvant injection. Simple randomization was used to assign the mice to a group.

All persons obtaining measurements and conducting the experiments and data analysis were aware of the group allocation throughout the experiment.

### 2.2. Vaccination

A 489-amino acid mosaic nucleoprotein (MNP) was produced and utilized as previously described (Creative BioMart, NY, USA; no catalogue number [Cat #] provided) [23]. Hen egg white ovalbumin grade V (OVA) was purchased from Sigma-Aldrich (St. Louis, MO, USA; Cat # A5503). Adjuplex (ADJ) was provided by the Vaccine Center at the National Institutes of Health (Bethesda, MD, USA; no Cat # provided). Cyclic dinucleotides (CdN) and glucopyranosyl lipid adjuvant (GLA; (Monophosphoryl Lipid A [Synthetic], PHAD™) were purchased from InvivoGen (San Diego, CA, USA; Cat # vac-nacga23s) and Avanti Polar Lipids, Inc. (Alabaster, AL, USA; Cat # 699800P), respectively.

Each cow received a 1 mL subcutaneous injection (left neck) containing 50 μg of MNP formulated in 0.9% sodium chloride (saline; purchased from Sigma-Aldrich [MO, USA]; Cat # S8776) with ADJ (10%) and either CdN (50 μg; *n* = 5) or GLA (50 μg; *n* = 4). Control animals received saline alone (*n* = 5). The adjuvant dosage was based on prior murine immunogenicity data and adjusted to account for the larger body mass and total vaccine volume required for cattle [8,9,15,23]. Booster immunizations were administered on days 21 and 91 following primary vaccination. Mice were briefly anesthetized with isoflurane and injected subcutaneously in the left hind footpad with 25 μL containing 15 μg of OVA formulated in saline with ADJ (5%) and either CdN (2.5 μg; *n* = 5) or GLA (2.5 μg; *n* = 5). Control mice received 15 μg of OVA in saline (*n* = 5) or saline alone (*n* = 3). Injections were performed as previously described [8]. Tissues were collected 48 h post-vaccination.

### 2.3. Enzyme-Linked Immunosorbent Assay (ELISA)

Blood was collected from the tail vein of all cattle prior to vaccination and biweekly thereafter, while milk samples were collected weekly. Serum and milk samples were submitted to the Wisconsin Veterinary Diagnostic Laboratory (WVDL) within a day of collection but were stored at 20 ℃ or 4 ℃ until submission, respectively. Antibodies against H5N1 nucleoprotein in serum and milk were quantified using a competitive ELISA performed by NAHLN-proficiency-tested WVDL personnel. The assay utilized the IDEXX Avian Influenza (AI) MultiS-Screen Antibody Test (IDEXX Laboratories, Westbrook, ME, USA; Cat # not provided), a NAHLN-approved assay. Non-negative results were confirmed by the National Veterinary Services Laboratories (NVSL).

### 2.4. Preparation of Mononuclear Cells

Peripheral blood mononuclear cells (PBMCs) were isolated from tail vein blood collected from cattle according to and as validated by the laboratory’s standard operating procedure (SOP) as follows. PBMCs were separated by density gradient centrifugation using Histopaque-1077 or Histopaque-1119 (Sigma-Aldrich, St. Louis, MO, USA; Cat #s 10771 and 11191, respectively). Residual red blood cells were lysed by ammonium-chloride-potassium (ACK) lysing buffer (Thermo Fisher Scientific, Grand Island, NY, USA; Cat # A10492-01) and incubated as previously described [8]. For murine studies, the draining left popliteal and distant right axillary lymph nodes (LNs) were harvested and incubated in 3 mL of filtered collagenase D (1 mg/mL; Roche/Sigma-Aldrich, MO, USA; Cat # 11088882001) for 30 min at 37 ℃. Following enzymatic digestion, lymph nodes were mechanically dissociated and passed through a 70 μm filter to generate single-cell suspensions.

### 2.5. Enzyme-Linked ImmunoSpot (ELISPOT)

Isolated PBMCs were enumerated, and an ELISPOT was performed with 5 × 10^5^ cells per well, utilizing an ELISPOT Plus: Bovine IFN-γ [HRP] kit (Mabtech Inc., Cincinnati, OH, USA; Cat # 3119-4HPW-2) according to the manufacturer’s instructions. Concanavalin A (Sigma-Aldrich, MO, USA; Cat # C2010) was included as a positive control. PBMCs were stimulated with two peptide pools (1 μg/mL per peptide), each consisting of 30 unique 14–16-mer peptides derived from the nucleoprotein of Influenza A/California/04/2009 (H1N1) pdm09. These peptides were selected from a library of 122 overlapping peptides spanning the full-length nucleoprotein (BEI Resources, Manassas, VA, USA). Pool 1 contained peptides 1–30, and Pool 2 contained peptides 31–60 (Table S1, Supplemental Materials). IFN-γ–producing spots were manually enumerated and quantified relative to unstimulated control wells (no peptide).

### 2.6. Flow Cytometry

Tissues were processed into single-cell suspensions according to the laboratory’s SOP as described above. Single-cell suspensions from lymph nodes were stained with Live/Dead Ghost Dye 780 (eBioscience, San Diego, CA, USA; Table S2, Supplemental Materials) diluted in phosphate-buffered saline (PBS; Cytiva, Logan, UT, USA; Cat # SH30256.02) for 30 min on ice. After washing, cells were incubated with anti-CD16/CD32 Fc block (Tonbo Biosciences, San Diego, CA, USA; Table S2, Supplemental Materials) diluted in fluorescence-activated cell sorting (FACS) buffer for 15 min on ice. FACS buffer is composed of 2% bovine serum albumin (GOLDBIO, St. Louis, MO, USA; Cat # A-420-500) and 0.1% sodium azide (Dot Scientific Inc., Burton, MI, USA; Cat # DSS24080-250) in PBS. Cells were then washed and stained for 30 min on ice with fluorochrome-conjugated antibodies (Table S2, Supplemental Materials) diluted in Brilliant Stain Buffer (BD Biosciences, San Diego, CA, USA; Cat # 566349). Following surface staining, cells were washed and fixed with 2% paraformaldehyde (Electron Microscopy Sciences, Hatfield, PA, USA; Cat # 15710-S) diluted in PBS for 15 min on ice. Samples were acquired on a BD FACSDiscover A8 (BD Biosciences, CA, USA) and analyzed using FlowJo v11 software (Tree Star, Ashland, OR, USA).

### 2.7. Statistical Analysis

All statistical assumptions were verified (including normality via the Shapiro–Wilk Test and QQ plots, as well as homogeneity of variances) in GraphPad Prism (version 10.6.0). Feed intake, milk yield, body weight, and temperature were analyzed using repeated measures ANOVA and further analyzed with pairwise *t*-tests with Benjamini–Hochberg (BH) correction in RStudio (version 2025.09.2+418; Posit Software, PBC). Area under the curve (AUC) analyses were performed in GraphPad Prism (version 10.6.0). Assessment of these parameters in relation to parity was analyzed using linear mixed model (LMM) and further analyzed with pairwise *t*-tests with Benjamini–Hochberg (BH) correction in RStudio (version 2025.09.2+418). For all of the analyses above, samples sizes of *n* = 5 were utilized for the control and ADJ + CdN groups, and *n* = 4 was utilized for the ADJ + GLA group. An LMM was selected because there was only one animal with a parity of 1 (birthed one offspring prior to the experiment and lactation). Otherwise, groups of parities 2, 3, and 4 had samples sizes of *n* = 4, 7, and 2, respectively. Feed intake values for one animal with a parity of 4 (and part of the ADJ + GLA group) were excluded from day 112 onward due to a physical injury that resulted in reduced feed intake.

ELISA and ELISPOT data were assessed for normality of titer values and repeated measures. ELISA data were analyzed at each time point using pairwise *t*-tests with BH correction in RStudio (version 2025.09.2+418) with *n* = 5, 5, and 4 for control, ADJ + CdN, and ADJ + GLA groups, respectively. Missing values were imputed using the group mean. Levene’s test was performed on ELISA data from the CdN group (*n* = 5) to assess homogeneity of variance. Number of spots of unstimulated T-cells were subtracted from number of spots of peptide-stimulated T-cells in each animal. This resultant number was log_2_-transformed. ELISPOT data were analyzed using one-way ANOVA followed by BH-adjusted pairwise *t*-tests at each time point in RStudio (version 2025.09.2+418). ELISPOT analysis was performed for control, ADJ + CdN, and ADJ + GLA groups with *n* = 5, 5, and 4, respectively. Lymph node flow cytometry data were analyzed by one-way ANOVA with multiple comparisons or (if assumptions were not met) Kruskal–Wallis and Dunn’s tests in GraphPad Prism (version 10.6.0). Analysis was performed using *n* = 5, 5, 5, and 3 for ADJ + CdN, ADJ + GLA, OVA, and saline groups of mice, respectively. There was no exclusion of mice or data from any of the analyses performed on the mouse study.

## 3. Results

### 3.1. Safety of Adjuvant Formulations in Cattle

**Figure 1:**
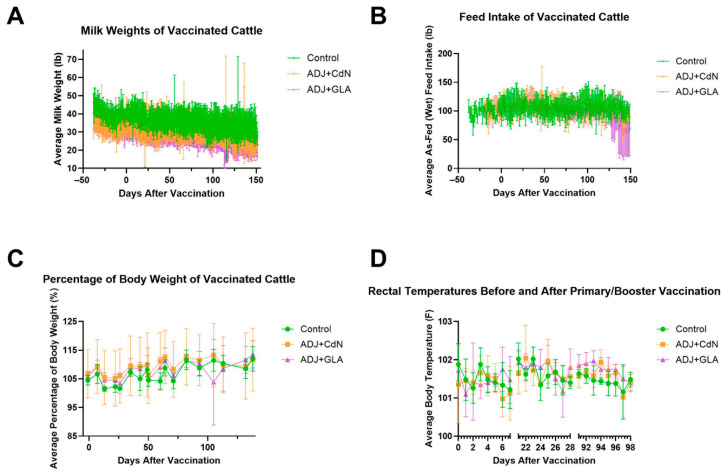
Safety assessment of vaccine formulations. Milk yield, feed intake, and body weight of experimental and control cattle were recorded twice daily, daily, and weekly, respectively. Rectal body temperature was measured on the day of vaccination or booster, prior to vaccination or booster, and 7 days post-immunization. (**A**) Mean milk yield of each group before and throughout the study (lb). (**B**) Mean as-fed (wet) feed intake of each group before and throughout the study (lb). (**C**) Mean body weight of each group before and throughout the study period (lb). (**D**) Mean rectal body temperature of each group measured prior to vaccination or booster, on the day of vaccination or booster, and 7 days post-immunization (°F).

Individual cattle were vaccinated subcutaneously in the left neck with ADJ + CdN + MNP (*n* = 5), ADJ + GLA + MNP (*n* = 4), or saline (control; *n* = 5). Following the primary vaccination (day 0), booster doses were administered on days 21 and 91. We did not use adjuvant-only controls because our previous work has shown that ADJ alone provided no protection or elicited NP-specific T-cell responses [23]. Vaccine safety was evaluated by monitoring milk yield (twice daily), feed intake (daily), body weight (weekly), and rectal temperature (on the day of vaccination and for 7 days thereafter). All measurements failed to reveal overall group differences; in other words, the vaccinated groups were not uniformly different from the control group across the entirety of the study. Three out of the four outcomes (feed intake, and milk and body weights) exhibited a transient decline throughout the study which was observed in all groups. These three outcomes also showed significant interactions between the groups and day, meaning there were differences in these physiological parameters between vaccinated groups and the control at specific time points during the study. The average feed intake of the ADJ + GLA group was significantly decreased compared to the control group on days 129 and 136 (*p* = 0.0315 and 0.0384, respectively) (Figure 1B). A transient decline in milk production was observed in both ADJ + GLA and ADJ + CdN groups on day-6 (6 days prior to vaccination) relative to baseline at primary vaccination and controls (*p* = 0.0317) (Figure 1A). Thereafter, milk production in the ADJ + CdN group remained comparable to controls. In contrast, additional significantly reduced milk yields were recorded in the ADJ + GLA group on days 10, 19, 28, and 53 compared to controls (*p* = 0.0416, 0.039, 0.0325, and 0.0305, respectively). Similarly, a transient increase in body weight was noted on day 60 in the ADJ + CdN group compared to controls (*p* = 0.0306); however, body weights remained stable across all groups at previous and subsequent time points (Figure 1C). Cattle were classified as febrile when rectal temperature exceeded 103 °F (normal cattle rectal temperature ranges from 99 to 101.5 °F). A single febrile episode (103.5 °F) occurred in one ADJ + CdN-vaccinated cow on day 22 (one day after the first booster); the temperature returned to <103 °F within 24 h and remained normal during the 7-day monitoring period. Despite this incident, there was no differential temperature effect at any time point (Figure 1D). No injection-site reactions were detected within 48 h following primary or booster vaccinations.

The relationship between these parameters and parity was also evaluated. Parity significantly interacted with time for both feed intake and milk production (*p* < 0.001), indicating that the trajectories of these parameters across the study period differed between parity groups. One animal with a parity of 4 had a markedly reduced milk yield compared to cows with parities of 2 and 3 on day 113 (*p* = 0.039). Cows with a parity of 4 had reduced feed intake compared to cows with a parity of 2 on days 78, 80, 85, and 87 and compared to cows with a parity of 3 on days 85, 87, and 93 (*p* < 0.05). Parity significantly influenced body weight throughout the study, with higher-parity animals maintaining greater body mass, consistent with known continued growth in dairy cattle. Rectal temperature was unaffected by parity at any time point.

### 3.2. Vaccine-Induced Serum Antibody Responses to Influenza Nucleoprotein

**Figure 2:**
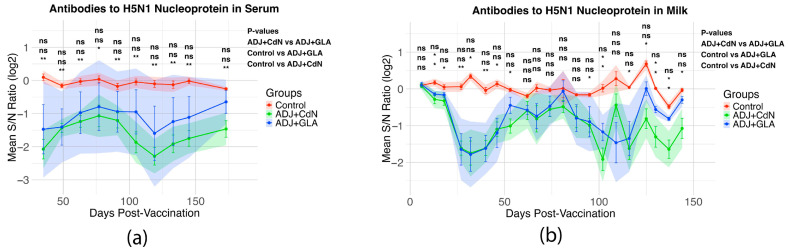
Antibody responses to H5N1 influenza nucleoprotein in serum and milk. Cattle were vaccinated on days 0, 21 (first booster), and 91 (second booster) with ADJ + CdN + MNP (CdN), ADJ + GLA + MNP (GLA), or saline (Control). Longitudinal plots depict mean antibody signal-to-noise (S/N) ratios measured by competitive ELISA against H5N1 influenza A nucleoprotein, shown on a log_2_ scale. Pairwise *t*-tests were performed for two-group comparisons. *, ** indicate statistical significance at *p* < 0.05 and *p* < 0.01, respectively; “ns” denotes not significant. Reported *p*-values correspond to CdN vs. GLA (top), Control vs. GLA (middle), and Control vs. CdN (bottom). Shaded ribbons represent 95% confidence intervals (CI). (**a**) Antibody responses in serum; (**b**) Antibody responses in milk.

Cattle were vaccinated with ADJ + CdN + MNP, ADJ + GLA + MNP, or saline (control) as described above. Serum samples were collected and processed individually biweekly, and sera antibodies were analyzed by competitive ELISA. Results were interpreted based on the signal-to-noise (S/N) ratio: samples were considered positive when S/N < 0.4, suspect when 0.4 ≤ S/N < 0.5, and negative when S/N ≥ 0.5. As early as 14 days after the first booster, cattle vaccinated with ADJ + CdN exhibited significantly higher antibody responses compared with controls (*p* < 0.001) (Figure 2). Antibody levels in the ADJ + CdN group remained significantly elevated relative to controls throughout the study period (*p* < 0.001, except *p* < 0.01 at day 49). Levene’s test confirmed homogeneity of variances between groups from days 35–173 (*p* = 0.8365), supporting the robustness of these comparisons. Collectively, these findings demonstrate that ADJ + CdN reliably elicits a sustained humoral response in cattle. Although antibody kinetics in the ADJ + GLA group paralleled those observed with the ADJ + CdN group, responses did not reach statistical significance relative to controls at any post-vaccination time point (*p* > 0.05), which suggest comparatively weaker or more variable immunogenicity. Both vaccinated groups exhibited a transient decline in antibody levels, reaching a nadir at day 77, followed by a secondary peak around day 110, consistent with recall responses after boosting. While direct comparisons between ADJ + CdN and ADJ + GLA did not reveal statistically significant differences in serum antibody titers (*p* > 0.05), the consistent separation of ADJ + CdN from controls indicates greater functional potency of this formulation. Importantly, antibody levels were comparable across groups prior to and on the day of primary vaccination (Supplemental Figure S1), confirming the absence of pre-existing H5N1-specific immunity and strengthening the conclusion that observed responses were vaccine-induced.

### 3.3. Vaccine-Induced Antibody Responses to Influenza Nucleoprotein in Milk

Competitive ELISA was performed weekly on individual milk samples to quantify influenza nucleoprotein-specific antibodies in milk. As above, results were interpreted based on the signal-to-noise (S/N) ratio: positive (<0.6), suspect (0.6 ≤ S/N < 0.7), and negative (≥0.7). Within 13 days of primary vaccination, cattle receiving ADJ + CdN exhibited significantly elevated antibody levels in milk compared to controls (*p* < 0.01) (Figure 2). A marked increase was observed by day 27 (*p* < 0.001), and antibody levels remained significantly higher than controls through day 62 (*p* < 0.01). Following the second booster (day 91), strong antibody responses were evident by day 95 (*p* < 0.01), with peaks at days 102 and 116. Elevated antibody levels in the ADJ + CdN group were sustained through day 144 (*p* < 0.01). Levene’s test confirmed homogeneity of variances from days 27–144 (*p* = 0.9812), supporting the robustness of these comparisons. In contrast, antibody levels in the ADJ + GLA group were largely comparable to controls throughout the study (*p* > 0.05), with the exception of isolated time points (days 13 and 130). Notably, antibody levels of the ADJ + GLA group remained significantly elevated relative to controls after day 130 (*p* < 0.01). Direct comparisons between ADJ + CdN and ADJ + GLA groups did not reveal statistically significant differences at any time point (*p* > 0.05). Importantly, antibody levels in milk did not differ among groups prior to or on the day of vaccination (Supplemental Figure S2), confirming the absence of pre-existing nucleoprotein-specific immunity.

### 3.4. Vaccine-Induced IFN-γ-Producing T-Cells in the Circulation

**Figure 3:**
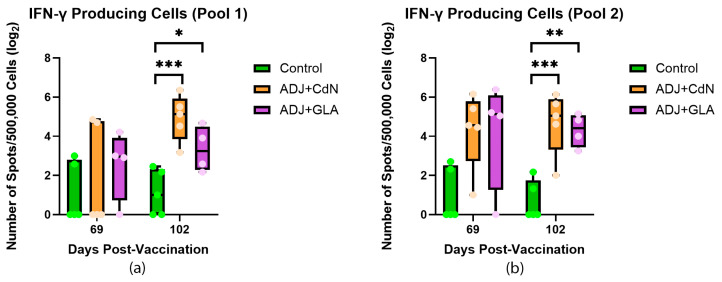
Influenza virus nucleoprotein-specific IFN-γ–producing cells in peripheral blood. Cattle were vaccinated on days 0, 21 (first booster), and 91 (second booster) with ADJ + CdN + MNP (CdN), ADJ + GLA + MNP (GLA), or saline (Control). Peripheral blood mononuclear cells (PBMCs) were collected on days 69 (48 days post-first booster) and 102 (11 days post-second booster). PBMCs were stimulated with two influenza nucleoprotein (NP) peptide pools, and IFN-γ–producing cells were quantified by ELISPOT assay. Dots are indicative of the number of IFN-γ–producing cells of individual animals on a log_2_ scale. The Control group was compared with the CdN and GLA groups using pairwise Welch’s *t*-tests. *, **, *** indicate statistical significance at *p* < 0.05, <0.01, and <0.001, respectively. (**a**) Peptide pool 1-stimulated IFN-γ-producing cells per well; (**b**) Peptide pool 2-stimulated IFN-γ-producing cells per well.

To quantify vaccine-induced T-cell responses to influenza NP, peripheral blood mononuclear cells (PBMCs) were stimulated with two pools of overlapping 14–16-mer peptides (30 peptides per pool) derived from the nucleoprotein of Influenza A/California/04/2009 (H1N1) pdm09 (Table S1). These peptides were used and validated against the MNP sequence in a previous study [21]. Peptide-specific IFN-γ-producing cells were quantified by ELISPOT. As shown in Figure 3, responses at day 69 following the first booster were variable among animals, although peptide-responsive T-cells were detectable in several vaccinated cattle. To improve the consistency of T-cell responses and increase the frequencies of memory T-cells, a second booster was administered at 91 days. By day 102 (11 days after the second booster), both peptide pools induced significantly greater numbers of IFN-γ-producing cells in vaccinated groups compared with controls (ADJ + CdN: *p* < 0.0001; ADJ + GLA: *p* < 0.01). Notably, responses in the ADJ + CdN group were more consistently elevated across animals. At day 145, peptide pool 1–specific IFN-γ responses remained significantly higher in the ADJ + CdN group compared with controls (*p* < 0.001), indicating persistence of vaccine-induced cellular immunity. Collectively, these findings demonstrate that both adjuvanted formulations elicit NP-specific T-cell responses in cattle, with ADJ + CdN promoting more robust and sustained cellular immunity.

### 3.5. Determining Adjuvant-Related Local and Systemic Innate Immune Responses

Data presented in Figure 2 and Figure 3 demonstrate that the combination adjuvants ADJ + CdN and ADJ + GLA differentially shape antibody and T-cell responses in cattle. To determine whether these differences reflect distinct early innate immune programs, we examined innate immune responses 48 h after vaccination in a murine model. C57BL/6 mice were vaccinated in the left footpad with saline or ovalbumin (OVA) formulated with ADJ + CdN or ADJ + GLA. Since we are only measuring innate immune responses and not T cell responses to vaccination, OVA was utilized as a well-characterized model antigen in the vaccine formulation. Footpad vaccination was selected to precisely compartmentalize the local inflammatory milieu within the ipsilateral draining lymph node relative to the systemic response in the distal contralateral lymph node. Draining (left popliteal) lymph nodes were analyzed to assess local responses, and contralateral (right axillary) lymph nodes were evaluated to assess systemic immune activation. Immune cell subsets were quantified by flow cytometry to determine the cellular composition of each lymph node.

**Figure 4:**
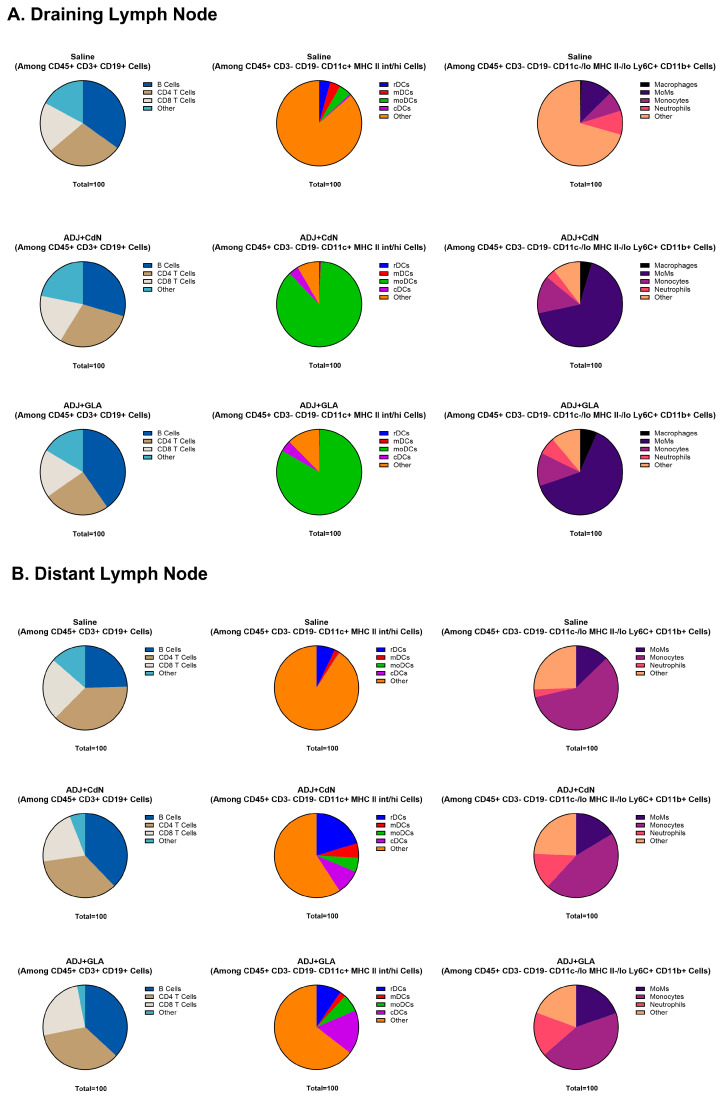
Innate cellular composition of lymph nodes in response to combination adjuvants. Groups of mice were vaccinated with ADJ + CdN + OVA, ADJ + GLA + OVA, OVA, or saline in the left footpad. Left popliteal (draining) and right axillary (distant) lymph nodes were collected 48 h after injection for flow cytometry. One-way ANOVA or Kruskal–Wallis and Dunn’s tests were conducted. (**A**) Percentage of cells from cell populations indicated in the left popliteal lymph node. (**B**) Percentage of cells from cell populations indicated in the right axillary lymph node.

Both ADJ + CdN and ADJ + GLA elicited robust local responses in the draining popliteal lymph node with similar cellular signatures. ADJ + CdN and ADJ + GLA vaccination triggered an increased frequency of monocyte-derived dendritic cells (moDCs; *p* < 0.005), conventional dendritic cells (cDCs; *p* < 0.001), and monocyte-derived macrophages (MoMs; *p* < 0.0001), as compared to saline (Figure 4A). Both adjuvant combinations were also associated with reduced frequency of resident dendritic cells (rDCs; *p* < 0.001), and migratory dendritic cells (mDCs; *p* < 0.01) compared to saline. In contrast, ADJ + GLA preferentially increased the frequency of macrophages (*p* < 0.01) compared to saline.

Systemically, both adjuvants induced immune activation in the contralateral axillary lymph node. ADJ + CdN and ADJ + GLA were associated with an increased frequency of moDCs (*p* < 0.0001) compared with saline. Otherwise, each adjuvant combination was associated with unique immunological signatures. ADJ + CdN vaccination resulted in a significantly increased frequency of rDCs (*p* < 0.05) and mDCs (*p* < 0.05) compared with saline and ADJ + GLA, indicating strong peripheral activation and/or trafficking (Figure 4B). ADJ + GLA vaccination increased the frequency of cDCs (*p* < 0.001) and neutrophils (*p* < 0.01) compared with saline, and increased CD8^+^ T-cells (*p* < 0.05) compared to ADJ + CdN, consistent with enhanced recruitment or expansion of innate antigen-presenting cell subsets. Together, these findings indicate that ADJ + CdN and ADJ + GLA differentially program early innate immune responses, with ADJ + CdN likely promoting activation of antigen-presenting cells locally and systemically and ADJ + GLA driving a more neutrophil-enriched and cytotoxic-skewed inflammatory profile. These distinct innate signatures might contribute to the qualitative differences observed in downstream adaptive immunity.

## 4. Discussion

Since the first reported detection of H5N1 HPAI virus in cattle, the B3.13 strain of the virus has spread to at least 2000 herds across 18 U.S. states, and long-term infections continue in at least two states [2,4]. While HPAI outbreaks continue to impact the poultry industry, a recent D1.1 infection at a dairy farm in Wisconsin was not epidemiologically linked directly to transmission from affected domestic poultry flocks two months earlier in the region or from interstate movement of infected cattle [24,25]. Similar to D1.1 strains found in Arizona and Nevada dairies in January 2025, no viral spread to adjacent dairies was noted in the dense dairy region of Wisconsin [25,26]. However, Wisconsin poultry did not see a significant spillover to nearby domestic poultry, which was observed on adjacent premises in Arizona. On the Wisconsin farm, no dairy cattle or farm workers showed any clinical signs, and the farm was identified through the National Milk Testing Strategy program [25]. Using PCR and ELISA on surveillance samples, it was estimated that the herd was infected ~6 weeks prior to detection. The isolate closely matched the domestic poultry isolate in the region months earlier, and the D1.1 strain has been the predominant strain in all U.S. flyways since 2024 [25]. Although the source of infection in this Wisconsin herd remains unclear, spillover from migratory birds is a plausible explanation. These observations underscore the complex and evolving ecology of H5N1 transmission at the wildlife–livestock interface. Preventing spillover events from wild birds to dairy cattle and preventing the rapid spread of viral strains like B3.13 from farm to farm may therefore represent a critical strategy to limit onward transmission to poultry and other susceptible mammals, including humans. In this context, development of effective vaccines to protect cattle against HPAI could serve as a proactive approach to interrupt transmission cycles and mitigate the broader One Health threat posed by H5N1. Because T-cell immunity is known to accelerate viral clearance and reduce viral replication and shedding, mitigating these parameters inherently decreases the environmental viral load. Consequently, we believe that reducing shedding in livestock is a critical step in lowering exposure pressure, thereby supporting protection at the human–animal interface.

Development of T-cell-based vaccines represents a promising strategy for universal influenza vaccine design [27,28,29]. We previously demonstrated that the carbomer-based nano-emulsion adjuvant ADJ elicits robust CD8 and CD4 T-cell responses by promoting dendritic cell-mediated cross-presentation [9]. In addition, we showed that influenza NP, a conserved internal antigen, formulated with a combination adjuvant consisting of ADJ and the TLR4 agonist GLA, induced potent CD4 and CD8 T-cell responses and conferred protection against heterologous influenza strains, including H1N1, H3N2, and H5N1, in mice [23,30]. Although innate immune signals are known to shape the magnitude and quality of adaptive T-cell responses, how distinct innate signaling pathways program T-cell immunity in a large outbred species such as cattle remains poorly defined. In the present study, we evaluated the safety and immunogenicity of two combination adjuvants designed to engage distinct innate pathways–TLR4 and STING–to determine how early innate cues influence vaccine-induced humoral and T-cell responses to the MNP protein in cattle.

To evaluate safety, we monitored milk production, body weight, feed intake, and rectal temperature throughout the study. Declines in milk yield observed during the experimental period were consistent with the expected physiological decrease over time and were not associated with vaccination. Although statistically significant differences in feed intake, milk production, and body weight were detected between vaccinated and control animals, these differences were most likely attributable to normal inter-animal biological variation, as vaccinated cattle did not exhibit reductions in body weight relative to their own pre-vaccination baselines. Moreover, no local adverse reactions–including swelling, erythema, raised lesions, or other signs of inflammation–were observed at the injection site for at least 48 h following vaccination. All measured parameters remained within normal age-appropriate ranges. Collectively, these findings indicate that the experimental vaccine formulations were well tolerated and safe for subcutaneous administration in cattle.

We also wanted to test if parity had any effect on feed intake, milk production, body weight, and body temperature to determine if parity could be a confounding variable in our study. Feed intake and milk production were affected by parity at very few time points. However, both parameters were similar across parities; thus, we suspect the differences in feed intake and milk production at the select time points were due to biological variance. The influence parity had on body weight was consistent with known continued growth in dairy cattle. Rectal temperature was unaffected by parity at any time point. Given these findings, parity does not appear to be a covariate in this study.

Because NP is an internal viral protein, NP-specific antibodies are unlikely to prevent influenza virus entry; however, they may contribute to antiviral immunity by supporting T-cell responses [23,27,28,30]. As a measure of vaccine-induced humoral immunity, we quantified NP-specific antibodies in serum and milk. Robust serum antibody responses were detected in all ADJ + CdN–vaccinated cattle and in a subset of ADJ + GLA–vaccinated animals within 14 days of the booster dose. Although antibody levels in the ADJ + CdN group remained significantly higher than in controls, substantial inter-animal variability limited the statistical significance of responses in the ADJ + GLA group at several time points. NP-specific antibodies were detectable in milk as early as 13 days post-vaccination in both CdN- and GLA-adjuvanted groups. Across the study period, ADJ + CdN–vaccinated cattle exhibited comparable or higher antibody titers in milk relative to ADJ + GLA–vaccinated animals. Thus, engagement of the STING pathway elicits robust and consistent antibody production in both serum and milk, as compared to the TLR4 pathway. Notably, antibody levels increased significantly in both serum and milk following the second booster (day 91), consistent with the induction of robust and durable B-cell memory, likely supported by vaccine-elicited T-cell help. The capacity of ADJ + CdN to generate strong systemic and mammary humoral responses suggests that incorporation of a surface antigen such as hemagglutinin could elicit virus-neutralizing antibodies and confer protection at both systemic and mucosal sites, including the mammary gland.

The differences in antibody kinetics between milk and serum may reflect the origin and isotype composition of antibodies within the mammary gland. Notably, NP-specific antibodies were detected earlier in milk (day 13) than in serum (day 35). Antibodies present in the mammary gland can arise from locally resident plasma cells and/or from transudation of circulating antibodies. Locally produced antibodies are often enriched for IgA, whereas serum responses are predominantly IgG. Because IgA can exhibit high functional avidity, particularly early in the response, it is possible that IgA in milk contributed to earlier detection in the competitive ELISA. This raises the possibility that vaccination induced mucosal IgA responses in the mammary gland, a hypothesis that warrants direct isotype-specific analysis in future studies. Alternatively, antibodies generated systemically early after vaccination may efficiently extravasate into the mammary gland and become concentrated in milk. Over time, antibody kinetics in serum and milk followed largely parallel trends. For example, S/N ratios began to decline–indicating increasing antibody levels–after day 21 in serum and day 18 in milk, and nadirs in antibody levels were observed at comparable time points (day 77 in serum and day 81 in milk). Collectively, these patterns suggest that most antibodies detected in milk likely derive from circulating antibodies, with possible contributions from local production.

Both T-cells and antibodies contribute to protection during primary influenza virus infection, and recent human studies have begun to define correlates of protective immunity. Pre-existing T-cell immunity has been associated with reduced disease severity during the 2009 H1N1 pandemic, and robust influenza-specific memory T-cell responses correlate with protection in humans [20,21,22,28,31]. Tissue-resident memory T-cells in the respiratory tract provide frontline immunity at sites of viral entry, whereas circulating memory T-cells offer a secondary layer of cell-mediated defense. In the present study, we quantified circulating NP-specific IFN-γ-producing memory T-cells by ELISPOT. Both ADJ + CdN and ADJ + GLA elicited type 1-polarized (TH1/TC1) NP-specific memory T-cell responses in peripheral blood. Importantly, these immune responses were detectable until at least day 145 post-vaccination. This T-cell response was especially pronounced after the second booster, which is suggestive of robust recall responses of memory T-cells. The 14–16-mer peptides used for PBMC stimulation likely activated both CD4^+^ and CD8^+^ memory T-cells; however, we did not directly determine the phenotype of NP-specific IFN-γ-producing cells in this study. Despite extensive optimization efforts, executing intracellular cytokine staining (ICCS) on the bovine peripheral blood samples presented persistent technical constraints. Based on our prior murine studies, we speculate that these vaccine formulations induce both memory CD4^+^ and CD8^+^ T-cell responses. Future work will incorporate optimized intracellular cytokine staining assays to define the phenotype and functional profile of vaccine-elicited memory T-cells in cattle. While the current study show that combination adjuvants elicited robust T-cell responses, lack of A-BSL-3 facilities for large animals precluded us from evaluating protective immunity to a H5N1 viral challenge. Future studies will assess whether MNP formulated with ADJ + CdN can provide heterosubtypic immunity to influenza viruses in cattle.

In this study, we found that ADJ + CdN elicited more consistent and robust antibody and T-cell responses compared to ADJ + GLA. To explore potential mechanisms underlying these differences, we performed complementary studies in mice to assess innate cellular responses 48 h after vaccination. Both combination adjuvants induced cellular recruitment in the draining lymph node as well as in the contralateral non-draining lymph node, indicating both local and systemic innate effects. ADJ + GLA and ADJ + CdN increased the frequencies of similar cellular profiles in the draining lymph node–including monocyte-derived DCs (moDCs), conventional DCs (cDCs), and monocyte-derived macrophages (MoMs)–with the exception of ADJ + GLA promoting greater frequencies of macrophages. In the non-draining lymph node, both adjuvant combinations induced increased frequencies of moDCs but had distinct cellular signatures otherwise. ADJ + CdN was associated with an increased frequency of rDCs and migratory DCs (mDCs), while ADJ + GLA contributed to an enhanced frequency of cDCs, CD8^+^ T-cells, and neutrophils. These patterns suggest that ADJ + GLA may drive a more pronounced pro-inflammatory response utilizing cross-presentation and neutrophilic inflammation, whereas ADJ + CdN induces a more balanced innate environment centered on type I (Th1) cellular immunity. Given that excessive inflammation can impair the formation and durability of memory T-cell responses, the moderated inflammatory milieu induced by ADJ + CdN may favor the development of higher-quality B- and T-cell immunity. Although the precise mechanisms accounting for the superior consistency of ADJ + CdN remain to be defined, our findings underscore the critical importance of adjuvant selection and highlight how distinct innate signaling pathways shape the magnitude and quality of vaccine-induced adaptive immunity. It is also important to note that adjuvant-antigen interactions can be antigen-specific, and the innate signatures observed with OVA + ADJ + CdN/GLA in mice may not recapitulate what happens with MNP + ADJ + CdN/GLA in cattle.

## Data Availability

The original contributions presented in this study are included in the article/Supplementary Material. Further inquiries can be directed to the corresponding author.

## Supplementary Materials

The following supporting information can be downloaded at https://www.mdpi.com/article/10.3390/v18060670/s1. Supplemental Figure S1: Pre-vaccination anti-H5N1 Nucleoprotein Serum Antibody levels. Supplemental Figure S2: Pre-vaccination anti-H5N1 Nucleoprotein Milk Antibody levels. Supplemental Table S1: Influenza Virus A/California/04/2009 (H1N1) pdm09 nucleocapsid protein peptides. Supplemental Table S2: List of antibodies in this study with dilution factors, clones, and catalogue or reference numbers used for flow cytometry.

## Abbreviations

The following abbreviations are used in this manuscript:

ACK	Ammonium-chloride-potassium
ADJ	Adjuplex
ANOVA	Analysis of variance
APHIS	Animal and Plant Health Inspection Service
AUC	Area under the curve
AWA	Animal Welfare Act
cDCs	Conventional dendritic cells
CdN	Cyclic dinucleotides
DCC	Dairy Cattle Center
DCs	Dendritic cells
ELISA	Enzyme-Linked Immunosorbent Assay
ELISPOT	Enzyme-Linked ImmunoSpot
FACS	Fluorescence-activated cell sorting
GLA	Glucopyranosyl lipid A
HA	Hemagglutinin
HPAI	Highly pathogenic avian influenza
IACUC	Institutional Animal Care and Use Committee
IAV	Influenza A virus
IFN-γ	Interferon-gamma
LMM	Linear mixed model
LN	Lymph node
LPS	Lipopolysaccharide
mDCs	Migratory dendritic cells
MNP	Mosaic nucleoprotein
moDCs	Monocyte-derived dendritic cells
MoMs	Monocyte-derived macrophages
NA	Neuraminidase
NAHLN	National Animal Laboratory Network
NP	Nucleoprotein
NVSL	National Veterinary Services Laboratories
OVA	Ovalbumin grade V
PBMCs	Peripheral Blood Mononuclear Cells
PBS	Phosphate-buffered saline
PHS	Public Health Service
rDCs	Resident dendritic cells
RdRp	RNA-dependent RNA polymerase
SARS-CoV-2	Severe Acute Respiratory Syndrome Coronavirus 2
STING	Stimulator of Interferon Genes
TLR4	Toll-like receptor 4
USDA	United States Department of Agriculture
WVDL	Wisconsin Veterinary Diagnostic Laboratory
