# Supplementary material for: Innate Pathway Selection Modulates Antibody and T-Cell Responses to Mosaic Influenza Nucleoprotein in Cattle
Source: Viruses. 2026 Jun 13;18(6):670. doi: 10.3390/v18060670 (PMC13307667; doi:10.3390/v18060670)
Supplement: Supplementary file 1 [file viruses-18-00670-s001.zip › viruses-4291814-supplementary.pdf]

Supplemental Figures

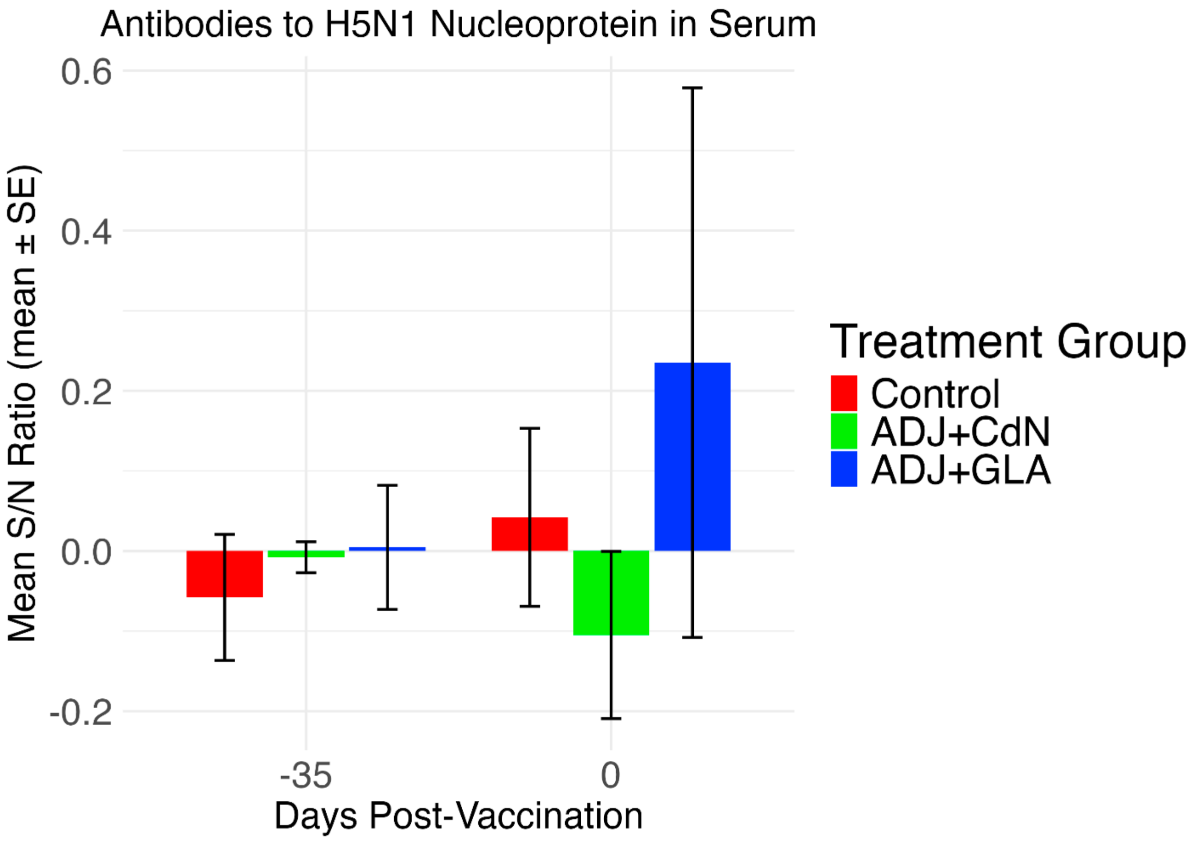

Figure S1. Pre-vaccination anti-H5N1 Nucleoprotein Serum Antibody levels. Mean serum antibody signal-to-noise (S/N) ratios within cattle groups on a log_2 scale. Pairwise *t*-test for two-group comparisons were conducted. On days -35 and 0, relative to vaccination, antibody levels were quantified in the serum from vaccinated cattle, ADJ+CDN+MNP (CdN) and ADJ+GLA+MNP (GLA).

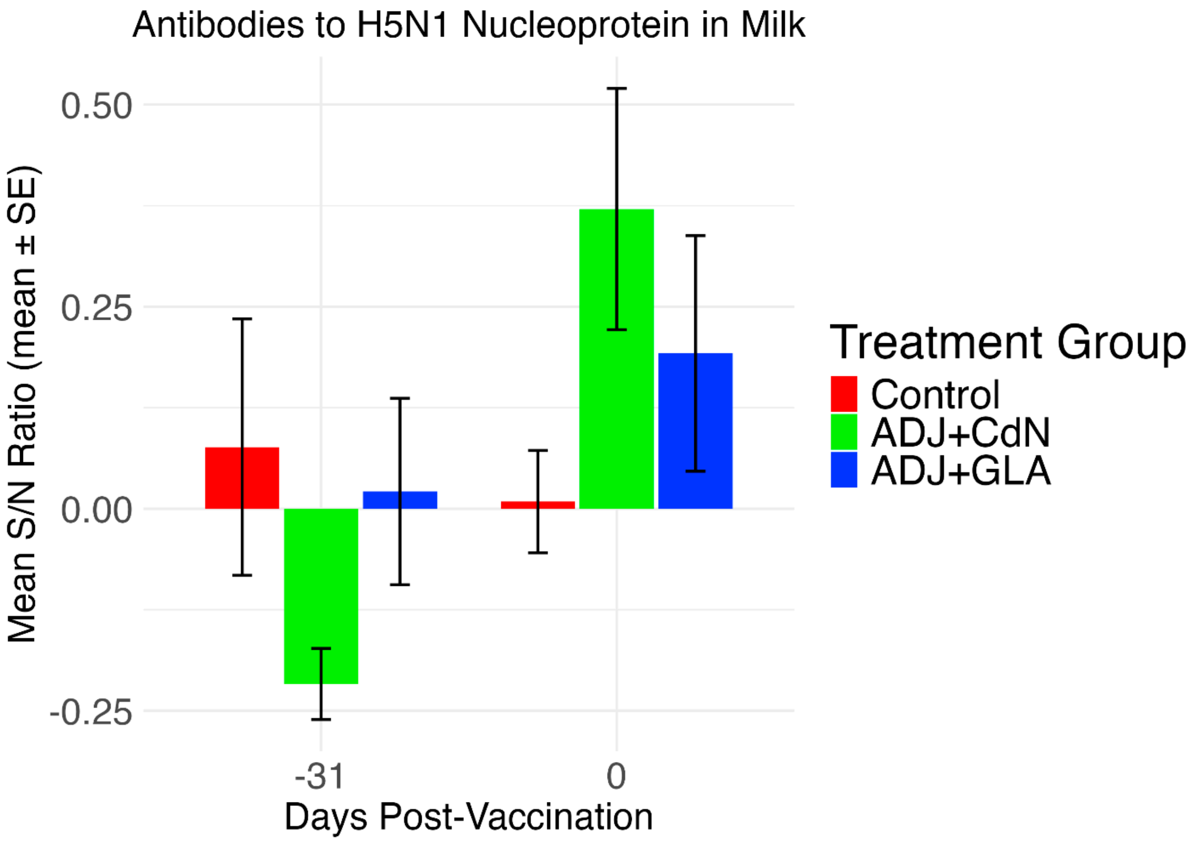

Figure S2. Pre-vaccination anti-H5N1 Nucleoprotein Milk Antibody levels. Mean milk antibody signal-to-noise (S/N) ratios within cattle groups on a log_2 scale. Pairwise *t*-test for two-group comparisons were conducted. On days -35 and 0, relative to vaccination, antibody levels were quantified in the milk from vaccinated cattle, ADJ+CDN+MNP (CdN) and ADJ+GLA+MNP (GLA).

Table S1. Influenza Virus A/California/04/2009 (H1N1) pdm09 nucleocapsid protein peptides. Peptide number with sequence, lot number, and molecular weight (amu) provided. Peptides were used to stimulate T-cells in ELISPOT. Pool 1 was composed of peptides 1–30 of 122, and peptide pool 2 was composed of 31–60 of 122. Table derived from “Certificate of Analysis for NR-18976” (BEI Resources, Manassas, VA).

**Peptide Number**	**Peptide Sequence**	**Lot Number**	**Molecular Weight (amu)**
1 of 122	1-MASQGTKRSYEQMET-15	LS1884	1747
2 of 122	5-GTKRSYEQMETGGER-19	LS1885	1729
3 of 122	9-SYEQMETGGERQDAT-23	LS1886	1702
4 of 122	13-METGGERQDATEIRA-27	LS1887	1664
5 of 122	17-GERQDATEIRASVGR-31	LS1888	1645
6 of 122	21-DATEIRASVGRMIGG-35	LS1889	1533
7 of 122	25-IRASVGRMIGGIGRF-39	LS1890	1590
8 of 122	29-VGRMIGGIGRFYIQM-43	LS1891	1698
9 of 122	33-IGGIGRFYIQMCTEL-47	LS1892	1701
10 of 122	37-GRFYIQMCTELKLSD-51	A4748	1804
11 of 122	41-IQMCTELKLSDYDGR-55	A4788	1772

12 of 122	45-TELKLSDYDGRLIQN-59	LS1895	1765
13 of 122	49-LSDYDGRLIQNSITI-63	LS1896	1708
14 of 122	53-DGRLIQNSITIERMV-67	LS1897	1745
15 of 122	57-IQNSITIERMVLSAF-71	LS1898	1722
16 of 122	61-ITIERMVLSAFDERR-75	LS1899	1836
17 of 122	65-RMVLSAFDERRNKYL-79	LS1900	1898
18 of 122	69-SAFDERRNKYLEEHPS-84	LS1901	1978
19 of 122	73-ERRNKYLEEHPSAGK-87	LS1902	1814
20 of 122	77-KYLEEHPSAGKDPKK-91	LS1903	1727
21 of 122	81-EHPSAGKDPKKTGGPI-96	LS1904	1619
22 of 122	85-AGKDPKKTGGPIYRR-99	LS1905	1644
23 of 122	89-PKKTGGPIYRRVDGK-103	LS1906	1672
24 of 122	93-GGPIYRRVDGKWMRE-107	LS1907	1820
25 of 122	97-YRRVDGKWMRELILY-111	LS1908	1998
26 of 122	101-DGKWMRELILYDKEE-115	LS1909	1925
27 of 122	105-MRELILYDKEEIRRV-119	LS1910	1963
28 of 122	109-ILYDKEEIRRVWRQA-123	LS1911	1975
29 of 122	113-KEEIRRVWRQANNGE-127	LS1912	1885
30 of 122	117-RRVWRQANNGEDATA-131	LS1913	1744
31 of 122	121-RQANNGEDATAGLTH-135	LS1914	1555
32 of 122	125-NGEDATAGLTHIMIW-139	LS1915	1629
33 of 122	129-ATAGLTHIMIWHSNL-143	LS1916	1665
34 of 122	133-LTHIMIWHSNLNDAT-147	LS1917	1766
35 of 122	137-MIWHSNLNDATYQRT-151	LS1918	1850
36 of 122	141-SNLNDATYQRTRALV-155	LS1919	1722
37 of 122	145-DATYQRTRALVRTGM-159	LS1920	1739
38 of 122	150-RTRALVRTGMDPRM-163	LS1921	1660
39 of 122	153-ALVRTGMDPRMCSLM-167	LS1922	1681
40 of 122	157-TGMDPRMCSLMQGST-171	LS1923	1615
41 of 122	161-PRMCSLMQGSTLPRR-175	LS1924	1733
42 of 122	165-SLMQGSTLPRRSGAA-179	LS1925	1532
43 of 122	169-GSTLPRRSGAAGAAV-183	LS1926	1371
44 of 122	173-PRRSGAAGAAVKGVG-187	LS1927	1354
45 of 122	177-GAAGAAVKGVGTIAM-191	LS1928	1274
46 of 122	181-AAVKGVGTIAMELIR-195	LS1929	1529
47 of 122	185-GVGTIAMELIRMIKR-199	LS1930	1688
48 of 122	189-IAMELIRMIKRGIND-203	LS1931-2	1773
49 of 122	193-LIRMIKRGINDRNFW-207	LS1932	1932
50 of 122	197-IKRGINDRNFWRGEN-211	LS1933	1875
51 of 122	201-INDRNFWRGENGRRT-215	LS1934	1891
52 of 122	205-NFWRGENGRRTRVAY-219	LS1935	1882
53 of 122	209-GENGRRTRVAYERMC-223	LS1936	1798
54 of 122	213-RRTRVAYERMCNILK-227	LS1937	1909
55 of 122	217-VAYERMCNILKGKFQ-231	LS1938	1800

56 of 122	221-RMCNILKGKFQTAAQ-235	LS1939	1709
57 of 122	225-ILKGKFQTAAQRAMM-239	LS1940	1694
58 of 122	229-KFQTAAQRAMMDQVR-243	LS1941	1781
59 of 122	233-AAQRAMMDQVRESRN-247	LS1942	1763
60 of 122	237-AMMDQVRESRNPGNA-251	LS1943	1676

Table S2. List of antibodies in this study with dilution factors, clones, and catalogue or reference numbers used for flow cytometry.

**Antibody (Dilution Factor)**	**Clone**	**Company**	**Catalogue/Reference Number**
Anti-mouse CD16/CD32 (1:100)	2.4G2	TONBO Biosciences	70-0161-U500
Anti-mouse CD4 (1:200)	RM4-5	Invitrogen	48-0042-82
Anti-mouse CD8a (1:200)	53-6.7	BD Horizon	563786
Biotin anti-mouse CD3 (1:100)	17A2	BioLegend	100243
Anti-mouse CD19 (1:200)	1D3	BD Pharmingen	553784
Streptavidin	---	BD Horizon	564666
Anti-mouse XCR1 (1:100)	ZET	BioLegend	148216
Anti-mouse CD45 (1:200)	30-F11	BD Horizon	563891
Anti-mouse B220 (1:200)	RA3-6B2	BD Horizon	563708
Anti-mouse I-A/I-E (1:600)	M5/114.15.2	BioLegend	107641
Anti-mouse CD11b (1:400)	M1/70	BD Horizon	563168
Anti-mouse CD11c (1:200)	HL3	BD Horizon	563735
Anti-mouse F4/80 (1:100)	BM8	eBioscience	69-4801-82
Anti-mouse Ly6G (1:200)	1A8	BioLegend	127607
Anti-mouse BST2 (1:200)	eBio927	Invitrogen	61-3172-82
Anti-mouse CD64 (1:200)	X54-5/7.1	BioLegend	139316
Anti-mouse Ly6c (1:300)	AL-21	BD Pharmingen	560593
Anti-mouse CD80 (1:200)	16-10A1	eBioscience	17-0801-82
Anti-mouse CD86 (1:200)	GL1	BD Horizon	565479
Anti-mouse CD103 (1:200)	M290	BD OptiBuild	758900
Viability Dye (1:1000)	---	TONBO Biosciences	13-0865-T100
